# Physical Inactivity and Cardiovascular Health in Aging Populations: Epidemiological Evidence and Policy Implications from Riyadh, Saudi Arabia

**DOI:** 10.3390/life15030347

**Published:** 2025-02-23

**Authors:** Abdulaziz M. Alodhialah, Ashwaq A. Almutairi, Mohammed Almutairi

**Affiliations:** 1 Department of Medical Surgical Nursing, College of Nursing, King Saud University, Riyadh 11545, Saudi Arabia; mohalmutairi@ksu.edu.sa; 2 School of Nursing & Midwifery, Monash University, Melbourne 3800, Australia; ashwaq.almutairi@monash.edu

**Keywords:** cardiovascular diseases, physical inactivity, older adults, risk factors, Saudi Arabia, public health

## Abstract

Background: Cardiovascular Diseases (CVDs) are the leading cause of morbidity and mortality globally, particularly among older adults. Physical inactivity, a modifiable risk factor, is strongly associated with the development and progression of CVD through its correlation with hypertension, diabetes, obesity, and hyperlipidemia. This study examines the association between physical inactivity and cardiovascular health among older adults in the Riyadh region, Saudi Arabia. Methods: A cross-sectional study was conducted on 168 participants aged 60 years and older attending a tertiary hospital in Riyadh. Data were collected using a structured questionnaire, the Physical Activity Scale for the Elderly (PASE), anthropometric measurements, and medical record reviews. Cardiovascular health indicators and risk factors were analyzed using descriptive statistics, chi-square tests, and multivariate logistic regression. Results: Participants with low physical activity levels had a higher prevalence of hypertension (78.2%), diabetes (64.4%), and obesity (51.3%) compared to those with high activity levels (41.8%, 28.7%, and 22.3%, respectively). Multivariate analysis showed that physical inactivity was significantly associated with an increased likelihood of CVD (adjusted OR: 1.98, *p* < 0.001), with hypertension and diabetes also being strong predictors. Conclusions: Physical inactivity is strongly correlated with adverse cardiovascular outcomes in older adults. Interventions targeting increased physical activity are essential to reducing the CVD burden. Community-based programs and policy-driven initiatives tailored to the Riyadh region’s specific environmental and cultural factors are crucial in promoting active lifestyles among aging populations.

## 1. Introduction

Cardiovascular diseases (CVDs) remain the leading cause of morbidity and mortality worldwide, particularly among aging populations [1]. As life expectancy increases globally, the burden of age-related health conditions, including heart disease, stroke, and peripheral artery disease, continues to rise [2]. Physical inactivity, a well-established modifiable risk factor for CVD, poses significant challenges for older adults, who often experience reduced mobility and engagement in regular physical activities due to physiological, psychological, and environmental barriers [3,4]. Addressing this issue requires a comprehensive understanding of the epidemiological evidence and effective policy interventions tailored to aging populations.

Epidemiological studies have consistently demonstrated a strong association between physical inactivity and increased risk of cardiovascular diseases [5]. Sedentary behavior contributes to the development of key CVD risk factors, including hypertension, hyperlipidemia, obesity, insulin resistance, and systemic inflammation [6]. According to data from the World Health Organization (WHO), insufficient physical activity is responsible for approximately 9% of premature mortality globally, with a significant proportion attributed to CVD [7]. In aging populations, physical inactivity exacerbates age-related declines in cardiovascular function, such as reduced arterial elasticity, impaired endothelial function, and decreased cardiac output [8].

Longitudinal cohort studies, such as the Framingham Heart Study, have provided robust evidence linking regular physical activity to reduced CVD incidence and mortality [9]. Older adults who engage in moderate-intensity aerobic exercise, such as walking or cycling, exhibit lower rates of coronary artery disease, heart failure, and ischemic stroke compared to their sedentary counterparts [10]. Furthermore, meta-analyses of randomized controlled trials (RCTs) have highlighted the cardioprotective effects of physical activity through improvements in lipid profiles, blood pressure, and glucose metabolism [11].

Despite the clear benefits of physical activity, adherence to exercise recommendations remains low among older adults [12]. A variety of barriers contribute to this trend, including physical limitations, such as arthritis, joint pain, or chronic illness, which reduce mobility and exercise capacity [13]. Psychological factors, such as fear of injury, lack of motivation, and depression, further discourage physical activity [14]. Additionally, social determinants of health, including low socioeconomic status, limited access to safe exercise environments, and lack of social support, compound the challenges faced by older individuals [15,16].

The COVID-19 pandemic further exacerbated physical inactivity among older populations due to widespread lockdowns, reduced access to fitness facilities, and increased social isolation. These challenges underscore the importance of targeted interventions to address the unique needs and circumstances of aging populations [17]. Epidemiological evidence provides a critical foundation for developing and implementing policies to promote physical activity and improve cardiovascular health among older adults [18]. Successful policy strategies should encompass individual, community, and societal levels, addressing both direct and indirect barriers to physical activity [19].

At the individual level, tailored exercise programs that account for physical and psychological limitations are essential. Initiatives such as supervised exercise classes, home-based exercise regimens, and telehealth interventions have shown promise in improving adherence and cardiovascular outcomes [20]. Programs like cardiac rehabilitation, which integrate physical activity with education and behavioral support, offer additional benefits for older adults with pre-existing CVD [21]. Community-level interventions play a crucial role in fostering environments that encourage physical activity [22]. The development of age-friendly urban spaces, including accessible parks, walking trails, and community centers, can help overcome environmental barriers [23]. Furthermore, community-based programs that provide group exercise opportunities and peer support can enhance social engagement and reduce feelings of isolation, which are common among older adults [24].

At the societal level, health promotion campaigns and policy initiatives aimed at increasing awareness of the benefits of physical activity are critical. Governments and public health organizations should prioritize funding for research on effective interventions and allocate resources to support physical activity programs targeting older adults [25]. In addition, collaboration between healthcare providers, policymakers, and community organizations is necessary to create a comprehensive framework for promoting cardiovascular health through physical activity [26].

Despite the wealth of epidemiological evidence, gaps remain in understanding the most effective strategies to promote physical activity among diverse aging populations. Future research should explore the impact of cultural, socioeconomic, and geographic factors on physical activity patterns and cardiovascular outcomes [27]. Additionally, the role of emerging technologies, such as wearable fitness trackers and virtual reality-based exercise programs, warrants further investigation [28]. Long-term studies are needed to assess the sustainability and cost-effectiveness of various interventions, as well as their potential to reduce healthcare costs associated with CVD [29]. Furthermore, research should examine the integration of physical activity promotion into routine healthcare for older adults, including the role of primary care providers in prescribing exercise and monitoring adherence [30].

Despite extensive research globally on the association between physical inactivity and cardiovascular disease, there remains a notable gap in understanding how these relationships manifest within the unique context of Saudi Arabia [31]. In particular, Riyadh’s extreme climatic conditions, characterized by high temperatures that limit outdoor physical activity, may significantly influence exercise behaviors. Additionally, cultural attitudes toward exercise—including gender-specific norms and societal expectations—differ markedly from those in Western or Asian settings. Moreover, variations in healthcare access and the design of urban infrastructure in Riyadh further differentiate this region from others studied in the literature [32]. Addressing these regional disparities is crucial, as they may affect both the prevalence of physical inactivity and its correlation with cardiovascular health outcomes. This study aims to fill this gap by specifically investigating the association between physical inactivity and CVD among older adults in Riyadh, thereby providing context-specific insights that can inform tailored public health policies and interventions.

### 1.1. Aim of the Study

This study aims to examine the association between physical inactivity and cardiovascular health among older adults in Riyadh, Saudi Arabia. The primary focus is on modifiable risk factors, including physical inactivity, obesity, and hypertension, while also acknowledging the role of non-modifiable factors such as age, gender, and genetic predisposition. By analyzing the interaction between these risk factors, this study seeks to provide a comprehensive understanding of how physical inactivity contributes to cardiovascular health outcomes in this specific population. Given the geographical and cultural uniqueness of Riyadh—where high temperatures, urban infrastructure, and cultural attitudes toward exercise may influence physical activity levels—this research provides region-specific insights that can inform targeted policy interventions and public health strategies.

### 1.2. Research Question

This study explores the association between physical inactivity and cardiovascular health in older adults in Riyadh, Saudi Arabia. To provide a structured analytical framework, the primary research question is divided into the following sub-questions:

1. What is the association between physical inactivity and key cardiovascular disease (CVD) risk factors (e.g., hypertension, diabetes, obesity) in older adults in Riyadh?
2. What are the demographic and socioeconomic determinants of physical inactivity among older adults in this population?
3. How do existing policy recommendations align with the specific barriers to physical activity in Riyadh, and what strategies can be proposed to address these barriers?

## 2. Materials and Methods

### 2.1. Study Design

This study utilized a cross-sectional design to explore the relationship between physical inactivity and cardiovascular health in older adults in the Riyadh region of Saudi Arabia. The design was chosen to provide a snapshot of the prevalence and impact of physical inactivity on cardiovascular outcomes in a specific population at a single point in time.

### 2.2. Setting

The study was conducted at a tertiary hospital in the Riyadh region, Saudi Arabia. The hospital is a major referral center, serving a diverse population and providing specialized medical services, including cardiology, geriatrics, and rehabilitation. The study population consisted of elderly patients attending outpatient and inpatient services within the hospital.

### 2.3. Sample and Sampling

The study sample consisted of 168 elderly individuals aged 60 years and above, recruited from a tertiary hospital in the Riyadh region of Saudi Arabia. These participants represented a diverse demographic, reflecting the population typically served by the hospital, including individuals from various socioeconomic and cultural backgrounds. The sample size was determined based on previous epidemiological studies exploring the association between physical inactivity and cardiovascular health, ensuring sufficient statistical power to detect meaningful associations.

### 2.4. Inclusion Criteria

Participants were eligible for inclusion in the study if they met the following criteria:

1. Age: Participants aged 60 years or older were selected to focus on the elderly population, where the risk of cardiovascular diseases and physical inactivity tends to be higher.
2. Medical Status: Individuals diagnosed with or at risk for cardiovascular diseases, as determined by their medical records or clinical evaluation, were included to ensure the study’s relevance to cardiovascular health.
3. Hospital Attendance: Only patients attending the hospital for routine medical care, follow-ups, or cardiovascular-related issues were considered.
4. Consent: Participants were required to provide informed consent. In cases where participants were unable to consent independently (e.g., due to mild cognitive impairment), consent was obtained from their legal guardians or caregivers.

### 2.5. Exclusion Criteria

Participants were excluded from the study if they met any of the following criteria:

1. Severe Cognitive or Communication Impairments: Individuals unable to comprehend the study procedures or respond to the questionnaire due to severe cognitive decline, dementia, or language barriers were excluded.
2. Terminal Illnesses: Patients with terminal conditions or those receiving palliative care were excluded, as their health status could significantly confound the study results.
3. Non-Compliance: Individuals unwilling or unable to complete the questionnaire or participate in anthropometric measurements were excluded.

### 2.6. Sampling Technique

A purposive sampling method was employed to recruit participants. This non-probability sampling approach was chosen to ensure the inclusion of individuals with specific characteristics relevant to the study objectives, namely older adults with a potential risk of cardiovascular diseases. Recruitment took place over a three-month period, during which research assistants identified eligible participants from outpatient clinics and inpatient wards within the hospital.

The recruitment process involved:

1. Screening: Medical records and patient appointment schedules were reviewed to identify potential participants who met the inclusion criteria.
2. Invitation: Eligible individuals were approached in person, and the study objectives and procedures were explained in detail.
3. Voluntary Participation: Participation was entirely voluntary, and individuals were given sufficient time to decide whether to join the study.

### 2.7. Justification for Sample Size

The sample size for this study was determined using power analysis, ensuring adequate statistical power to detect significant associations between physical inactivity and cardiovascular disease (CVD) risk factors. Based on prior epidemiological studies investigating the relationship between physical activity and CVD, an estimated effect size of 0.30 was used. Using G*Power 3.1 software, a minimum sample of 150 participants was required to achieve 80% power at a 95% confidence level (α = 0.05). To account for potential non-response and incomplete data, a 10% adjustment was made, leading to a final target sample of 168 participants.

A purposive sampling method was employed to ensure the inclusion of older adults at risk for CVD while maintaining diversity in socioeconomic backgrounds. The study specifically recruited participants from a tertiary hospital in Riyadh, which may introduce selection bias; however, this setting allowed for access to individuals with varying degrees of cardiovascular health conditions, enabling a focused examination of the relationship between physical inactivity and CVD risk factors.

### 2.8. Data Collection Tools

To comprehensively assess the relationship between physical inactivity and cardiovascular health in older adults, several data collection tools were utilized. These tools were carefully selected and validated to ensure accuracy, reliability, and relevance to the study’s objectives. The tools included a structured questionnaire, the Physical Activity Scale for the Elderly (PASE), medical record reviews, and anthropometric measurements.

#### 2.8.1. Structured Questionnaire

A pre-validated structured questionnaire was developed to gather detailed information about the participants’ demographic and lifestyle characteristics, as well as their medical and family history. The questionnaire consisted of the following sections:

- Demographic Information: Questions on age, gender, marital status, education level, occupation (if applicable), and socioeconomic status.
- Lifestyle Factors: Inquiries about dietary habits, smoking history, alcohol consumption, and sleep patterns.
- Medical History: Questions on participants’ past and current medical conditions, particularly related to cardiovascular diseases (e.g., hypertension, hyperlipidemia, diabetes, and prior cardiovascular events such as myocardial infarction or stroke).
- Family History: Information on the prevalence of cardiovascular diseases and related conditions among first-degree relatives.

The questionnaire was administered in both Arabic and English to accommodate the linguistic needs of participants. For those unable to read or write, trained research assistants conducted face-to-face interviews to complete the questionnaire.

#### 2.8.2. Physical Activity Scale for the Elderly (PASE)

The PASE is a widely used and validated tool for assessing physical activity levels in older adults. It provides a comprehensive measure of the frequency, duration, and intensity of physical activities performed over the past week. The scale covers three main categories of physical activity:

- Leisure-Time Activities: Walking, swimming, dancing, and other recreational activities.
- Household Activities: Light and heavy housework, gardening, and yard work.
- Occupational Activities: Physical tasks associated with any part-time or volunteer work.

Participants were asked to report the time spent on each activity, and the PASE score was calculated based on a weighted scoring system. Higher scores indicated greater physical activity levels.

The Physical Activity Scale for the Elderly (PASE) is a widely used and validated tool for assessing physical activity levels in older adults. It has been validated in various populations, including studies in Western and Asian contexts, demonstrating good reliability and validity in measuring activity levels in elderly populations [33,34]. The PASE questionnaire was deemed appropriate for use in this study due to its comprehensive assessment of different activity domains, including leisure, household, and occupational activities, which align with common lifestyle patterns among older adults in Riyadh.

To ensure cultural and contextual applicability:

1. A pilot test was conducted with 15 older adults in Riyadh to assess comprehension and clarity. Minor adjustments were made in wording to reflect local activities, such as including references to indoor walking in shopping malls—a common alternative to outdoor exercise due to extreme weather conditions.
2. Bilingual administration: The PASE was offered in both English and Arabic, with trained research assistants available to clarify questions for participants with literacy challenges.
3. Expert review: The adapted questionnaire was reviewed by local geriatric and public health specialists to ensure its relevance to the Saudi Arabian context.

#### 2.8.3. Medical Record Review

Relevant clinical data were extracted from participants’ electronic medical records to obtain objective measures of cardiovascular health. This included:

- Blood Pressure: Most recent systolic and diastolic blood pressure readings.
- Lipid Profile: Levels of total cholesterol, low-density lipoprotein (LDL), high-density lipoprotein (HDL), and triglycerides.
- Blood Glucose Levels: Fasting blood glucose and HbA1c readings for participants with or without diabetes.
- Body Mass Index (BMI): Pre-recorded height and weight measurements used to calculate BMI.
- Cardiovascular Diagnoses: Documentation of any diagnosed cardiovascular conditions (e.g., coronary artery disease, heart failure, or arrhythmias).

The medical records provided a reliable source of data for identifying participants’ cardiovascular risk factors and health status.

#### 2.8.4. Anthropometric Measurements

Anthropometric data were collected to assess participants’ physical health and obesity-related risk factors. Standardized techniques were employed to ensure consistency and accuracy:

- Weight and Height: Measured using a calibrated digital scale and stadiometer, respectively. BMI was calculated as weight in kilograms divided by height in meters squared (kg/m^2^).
- Waist Circumference: Measured at the midpoint between the lower rib and the iliac crest using a non-stretchable measuring tape. This measurement was used to assess central obesity, a critical risk factor for cardiovascular diseases.
- Hip Circumference: Taken at the widest part of the hips to calculate the waist-to-hip ratio, another indicator of cardiovascular risk.

Each measurement was recorded twice, and the average value was used to ensure accuracy.

### 2.9. Data Collection Procedure

The data collection process was carefully designed to ensure the accuracy and reliability of the information gathered while minimizing the burden on participants. The procedure was conducted in several sequential steps:

### 2.10. Recruitment of Participants

Potential participants were identified from outpatient and inpatient lists at the tertiary hospital. Trained research assistants approached individuals who met the inclusion criteria during their hospital visits. The study’s purpose, objectives, and procedures were explained to the participants, highlighting the voluntary nature of their involvement. Participants were given an opportunity to ask questions, and informed consent was obtained either directly from the participant or, in cases of limited capacity, from a legally authorized representative.

### 2.11. Administration of the Structured Questionnaire

Once consent was obtained, the research assistants conducted face-to-face interviews using a pre-validated structured questionnaire. This step was essential for collecting demographic information such as age, gender, education level, and socioeconomic status. In addition, participants were asked about their lifestyle factors, including physical activity patterns, dietary habits, and smoking history. To accommodate participants with literacy difficulties, the research assistants provided verbal explanations and ensured that the questions were clearly understood.

### 2.12. Assessment of Physical Activity

To quantify physical activity levels, the Physical Activity Scale for the Elderly (PASE) was administered. Physical activity levels in this study were categorized using the Physical Activity Scale for the Elderly (PASE), a widely used and validated tool for assessing physical activity in older adults. The PASE score is a composite measure derived from self-reported engagement in leisure-time, household, and occupational activities over the past week, weighted by intensity and frequency.

Participants were classified into three activity levels based on their total PASE score distribution:

- Low physical activity: PASE score ≤ 60 (sedentary lifestyle, minimal activity)
- Moderate physical activity: PASE score 61–100 (occasional structured activity, moderate household chores)
- High physical activity: PASE score > 100 (frequent structured exercise, active daily routines)

Sarcopenia, a loss of muscle mass and strength common in older adults, is a critical factor influencing physical activity levels.

- Handgrip strength measurement (via a dynamometer)
- Gait speed testing (as a functional mobility indicator)
- Muscle mass estimation (via bioelectrical impedance analysis or DXA scans)

### 2.13. Anthropometric Measurements Procedure

While Body Mass Index (BMI) is widely used as an indicator of obesity and cardiovascular risk, it has recognized limitations, particularly in older adults. BMI does not distinguish between fat mass and muscle mass, making it an inadequate measure of body composition, especially in populations prone to sarcopenia (age-related muscle loss). A more precise approach would involve using Bioelectrical Impedance Analysis (BIA) or dual-energy X-ray absorptiometry (DXA) to assess body fat percentage and muscle mass. However, bioimpedance analysis was not used in this study due to logistical constraints, including the availability of specialized equipment in the hospital setting and the feasibility of conducting such assessments on a large sample of elderly participants. Future studies should integrate objective body composition assessments to provide a more accurate evaluation of obesity and its relationship with cardiovascular health.

Additionally, this study did not include a detailed analysis of nutritional habits, such as protein intake, micronutrient consumption, or dietary patterns, which are important factors in cardiovascular health and physical activity levels. Poor protein intake, for example, can accelerate muscle loss, reducing physical activity capacity and increasing CVD risk. Similarly, deficiencies in key micronutrients such as vitamin D, calcium, and omega-3 fatty acids can impact cardiovascular function. The exclusion of dietary factors represents a limitation of the study, as diet plays a critical role in both physical activity engagement and cardiovascular outcome

### 2.14. Medical Record Review Procedure

To gather clinical data related to cardiovascular health, the research team accessed participants’ electronic medical records with the appropriate authorization. Data on key cardiovascular indicators such as blood pressure, lipid profiles, fasting blood glucose levels, and body mass index (BMI) were extracted. This step provided an objective assessment of participants’ cardiovascular risk factors and overall health status.

### 2.15. Quality Control Measures

Throughout the data collection process, stringent quality control measures were implemented to ensure the reliability and validity of the data. Each day, completed questionnaires and measurement records were reviewed by the research coordinator to identify any missing or inconsistent information. When discrepancies were noted, the research assistants revisited participants promptly to clarify or correct the data. Additionally, regular team meetings were held to address challenges, provide feedback, and reinforce adherence to the standardized data collection protocol.

### 2.16. Data Analysis

Data analysis was conducted using the Statistical Package for the Social Sciences (SPSS) version 26 to ensure a thorough examination of the study’s findings. Descriptive statistics were used to summarize participants’ demographic characteristics, levels of physical activity, and cardiovascular health indicators. These included frequencies and percentages for categorical variables (e.g., gender, smoking status) and means with standard deviations for continuous variables (e.g., age, BMI, blood pressure).

To investigate the relationship between physical activity levels and cardiovascular risk factors, chi-square tests were employed for categorical variables, while independent t-tests and one-way ANOVA were used to compare mean cardiovascular health indicators (e.g., cholesterol levels, blood glucose) across different physical activity categories (low, moderate, high). Post hoc tests were performed when significant differences were found in ANOVA to pinpoint specific group differences.

For a more in-depth analysis, multivariate logistic regression was conducted to identify significant predictors of cardiovascular diseases. This allowed for adjusting potential confounding variables such as age, gender, comorbidities, and socioeconomic status, providing a clearer understanding of the relationship between physical inactivity and cardiovascular health outcomes. A *p*-value of less than 0.05 was considered statistically significant, ensuring the reliability and validity of the results. Data were cross-checked to ensure consistency and accuracy before analysis.

### 2.17. Data Normality

Before conducting ANOVA and regression analyses, data normality was assessed to ensure the appropriateness of parametric statistical tests. Normality was evaluated using the bpiro-Wilk and Kolmogorov-Smirnov tests, with results indicating that variables such as BMI, blood pressure, lipid profiles, and blood glucose levels did not perfectly follow a normal distribution (*p* < 0.05). Additionally, visual inspection of histograms, Q-Q plots, and boxplots was conducted to assess distribution patterns and detect potential outliers. Skewness and kurtosis values were also examined, with all variables falling within an acceptable range (±2), suggesting that deviations from normality were moderate. Where necessary, log transformations were applied to variables with higher skewness, such as blood glucose levels, to improve normality.

For post hoc comparisons in ANOVA analyses, Tukey’s Honestly Significant Difference (HSD) test was used for normally distributed variables to identify significant differences between physical activity groups. For non-normally distributed variables, the Games–Howell post hoc test was employed, as it does not assume equal variances and is suitable for heteroscedastic data. These post hoc tests were conducted for all key comparisons, ensuring a robust analysis of differences between physical activity levels and cardiovascular risk factors.

### 2.18. Path Analysis Details

Path analysis was conducted to examine the direct and indirect associations between physical inactivity and cardiovascular disease (CVD) while considering key mediating factors such as hypertension, diabetes, and obesity [35,36]. The model assumes linear relationships among variables, with physical inactivity as the primary predictor influencing intermediate variables, which in turn impact CVD risk. To ensure model robustness, multicollinearity was checked using variance inflation factors (VIF), and key confounders such as age, gender, and socioeconomic status were controlled. The structural model was designed to assess both direct effects (physical inactivity → CVD) and indirect effects through obesity, hypertension, and diabetes as mediators.

Maximum likelihood estimation (MLE) was used to estimate path coefficients in AMOS 26.0, and mediation effects were tested using bootstrapping with 5000 resamples, providing bias-corrected confidence intervals (CIs) for indirect effects. The Sobel test was also employed to verify mediation significance. Model fit was evaluated using standard indices, including the chi-square test (χ^2^/df < 5 for acceptable fit), Comparative Fit Index (CFI ≥ 0.90), Root Mean Square Error of Approximation (RMSEA < 0.08), and Standardized Root Mean Square Residual (SRMR < 0.05). Path analysis was conducted using Structural Equation Modeling (SEM) in AMOS 26.0 to assess the direct and indirect effects of physical inactivity on Cardiovascular Disease (CVD). Mediation effects were tested using the bootstrapping method with 5000 resamples, providing bias-corrected Confidence Intervals (CIs) to determine the significance of indirect pathways. The Sobel test was also applied to further validate the mediation effects. Model fit was assessed using standard indices, including the chi-square test (χ^2^/df), Comparative Fit Index (CFI), Root Mean Square Error of Approximation (RMSEA), and Standardized Root Mean Square Residual (SRMR).

### 2.19. Ethical Considerations

Ethical approval for this study was obtained from the Institutional Review Board (IRB) at King Saud University (Reference Number: 25-006). All procedures were conducted in compliance with the ethical principles outlined in the Declaration of Helsinki.

Informed consent was obtained from all participants, ensuring they understood the study purpose, procedures, potential risks, and benefits. Confidentiality and anonymity were maintained by assigning unique identification codes to participants and securely storing data in password-protected files.

## 3. Results

Table 1 summarizes the demographic characteristics of the 168 participants. The majority (70.6%) were aged between 60 and 69 years, with a relatively balanced gender distribution (54.2% male and 45.8% female). Educational attainment varied, with approximately 42.5% having completed high school, and nearly half of the participants reported a household income below SAR 10,000. No significant demographic differences were observed between the physical activity groups.

Physical activity levels were assessed using the Physical Activity Scale for the Elderly (PASE). Based on the total PASE scores, participants were categorized into three groups: low (≤60), moderate (61–100), and high (>100) physical activity. As shown in Table 2, the low activity group (51.8% of participants) had a mean PASE score of 42.7, with an average walking duration of 15.2 min per day and only 12.3% reporting engagement in vigorous exercise. In contrast, the high activity group (19.0% of participants) exhibited a mean PASE score of 120.3, an average walking duration of 52.1 min per day, and 58.7% reported engaging in vigorous exercise. These findings indicate a clear dose–response relationship: “higher physical activity levels” are characterized by a greater frequency, longer duration, and increased intensity of exercise.

Table 3 shows the prevalence of key cardiovascular risk factors by physical activity level. The low activity group had significantly higher prevalence rates of hypertension (78.2%, *p* < 0.001), diabetes (64.4%, *p* < 0.001), and obesity (51.3%, *p* < 0.001) compared to the high activity group (hypertension: 41.8%; diabetes: 28.7%; obesity: 22.3%). Similarly, hyperlipidemia was more prevalent among participants with low physical activity (69.0%) than among those with high physical activity (35.4%), with differences reaching statistical significance (*p* < 0.001). A higher prevalence of a history of stroke and coronary artery disease was also observed in the low activity group (12.6% and 28.7%, respectively) compared to the high activity group (4.3% and 9.8%, respectively; *p* < 0.05 and *p* < 0.001, respectively).

Table 4 presents the mean cardiovascular health indicators across the three physical activity groups. The low activity group demonstrated significantly higher mean systolic blood pressure (148.7 ± 9.4 mmHg, *p* < 0.001), diastolic blood pressure (92.3 ± 7.1 mmHg, *p* < 0.001), total cholesterol (236.5 ± 18.3 mg/dL, *p* < 0.001), LDL cholesterol (157.3 ± 14.8 mg/dL, *p* < 0.001), and blood glucose levels (161.7 ± 21.4 mg/dL, *p* < 0.001) compared to the high activity group, which had mean systolic and diastolic blood pressures of 125.4 ± 7.2 mmHg and 80.1 ± 5.6 mmHg, respectively. Post-hoc analyses were conducted using Tukey’s HSD test for normally distributed variables, confirming that differences between groups were statistically significant.

Multivariate logistic regression was used to assess predictors of cardiovascular disease, with adjustments for potential confounders. As shown in Table 5, physical inactivity was significantly associated with an increased likelihood of cardiovascular disease (adjusted OR: 1.98; 95% CI: 1.53–2.56; *p* < 0.001). Other significant predictors included hypertension (adjusted OR: 1.73; 95% CI: 1.34–2.23; *p* < 0.001), diabetes (adjusted OR: 1.52; 95% CI: 1.21–1.91; *p* < 0.001), and obesity (adjusted OR: 1.41; 95% CI: 1.12–1.79; *p* < 0.001). Smoking was also a modest predictor (adjusted OR: 1.29; 95% CI: 0.98–1.68; *p* = 0.045). The regression model explained approximately 45% of the variance in cardiovascular disease risk (R^2^ = 0.45), indicating moderate model strength.

**Figure 1:**
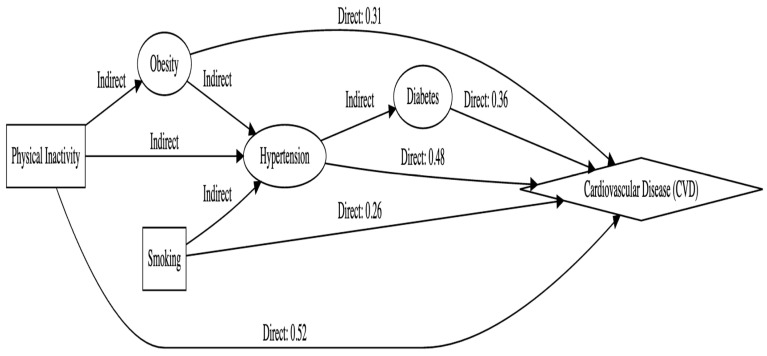
The path analysis model.

Figure 1 presents a path analysis model examining the direct, indirect, and total effects of various predictor variables on cardiovascular disease (CVD). Physical inactivity demonstrates the strongest total effect on CVD (0.73), with a substantial direct effect (0.52) and an indirect effect (0.21) mediated through obesity and hypertension. Hypertension follows closely, with a total effect of 0.63, of which 0.48 is direct and 0.15 is mediated via diabetes. Diabetes and obesity exhibit moderate total effects on CVD (0.48 and 0.49, respectively), with their indirect effects primarily channeled through other risk factors such as hypertension. Smoking has the weakest total effect on CVD (0.35), with most of its impact attributed to a direct effect (0.26) and a smaller indirect effect (0.09) through hypertension. All effects are statistically significant (*p* < 0.05), underscoring the importance of addressing these modifiable risk factors, particularly physical inactivity and hypertension, in CVD prevention strategies.

## 4. Discussion

The findings of this study reveal a strong relationship between physical inactivity and cardiovascular health among older adults in the Riyadh region. The results align with global evidence indicating that physical inactivity is a significant modifiable risk factor for cardiovascular diseases (CVD).

Participants with low physical activity levels exhibited the highest prevalence of cardiovascular risk factors, including hypertension, diabetes, obesity, and hyperlipidemia. This is consistent with previous studies that identify sedentary behavior as a major contributor to cardiovascular morbidity and mortality [6,37]. The path analysis further confirms that physical inactivity has the strongest total effect on CVD, highlighting its direct impact and indirect effects through other risk factors such as obesity and hypertension. These findings underscore the necessity of promoting physical activity to mitigate CVD risk in aging populations [38,39].

A clear dose-response relationship was observed, with participants in the high-activity group demonstrating significantly better cardiovascular profiles than those in the low-activity group. Higher physical activity levels were associated with improved lipid profiles, lower blood pressure, and reduced blood glucose levels. This aligns with meta-analyses demonstrating that even moderate increases in physical activity can lead to substantial improvements in cardiovascular health [40,41]. These results emphasize the importance of encouraging at least moderate physical activity, such as brisk walking, to reduce cardiovascular risk in older adults.

Demographic and socioeconomic factors also played a crucial role in influencing physical activity levels. Older participants, those with lower education levels, and individuals from lower-income households were more likely to exhibit low physical activity levels. These findings align with previous research indicating that socioeconomic disparities significantly affect health behaviors and outcomes [42,43]. Tailored interventions that address these disparities, such as community-based exercise programs and financial support for physical activity resources, are essential to promote equitable health outcomes [44,45].

The study also highlighted several psychological and social barriers to physical activity, such as fear of injury, lack of motivation, and social isolation. These barriers were particularly pronounced among participants in the low-activity group, reflecting findings from other studies on older adults [46]. Social determinants of health, including access to safe environments and supportive social networks, are critical in addressing these challenges [47]. Community-level interventions that foster social engagement, such as group exercise programs, may help overcome these barriers and encourage sustained physical activity [48,49].

The findings have important policy implications, particularly for public health strategies aimed at reducing the burden of CVD in older adults. Comprehensive policies targeting physical inactivity should operate at multiple levels—individual, community, and societal. At the individual level, integrating physical activity counseling into routine healthcare, especially in primary care settings, could increase awareness and motivation among older adults [50,51]. Community-level initiatives, such as the development of age-friendly urban spaces and the provision of free or subsidized exercise programs, can create an environment conducive to physical activity [52,53].

At the societal level, public health campaigns emphasizing the benefits of physical activity and the risks of inactivity are crucial. Additionally, partnerships between healthcare providers, policymakers, and community organizations can enhance the reach and effectiveness of these interventions [54,55].

The results of this study are comparable to findings from other regions, reinforcing the global relevance of physical activity in CVD prevention. For instance, the Framingham Heart Study demonstrated similar associations between physical activity and reduced CVD incidence [56]. However, unique cultural and environmental factors in the Riyadh region, such as high temperatures and limited walkable urban spaces, may pose additional challenges to physical activity [57,58]. These contextual factors must be considered when designing interventions tailored to local populations.

A notable strength of this study is its focus on the unique socio-environmental context of Riyadh. High ambient temperatures, which can persist for much of the year, severely limit opportunities for outdoor exercise [59]. Furthermore, the city’s built environment often lacks age-friendly infrastructure, such as safe walking paths and accessible recreational centers, which are crucial for encouraging regular physical activity among older adults. Cultural factors also influence exercise habits; traditional norms may restrict women’s participation in public exercise, necessitating gender-sensitive approaches [60,61,62]. In light of these challenges, policy recommendations specific to Saudi Arabia should include the development of indoor recreational facilities, subsidized fitness programs for older adults, and urban planning initiatives that incorporate age-friendly and gender-sensitive designs [63,64]. Tailored public health campaigns that address local cultural attitudes and promote safe, accessible exercise environments are equally important.

While this study provides valuable insights, further research is needed to explore culturally appropriate interventions to increase physical activity among older adults in the region. Additionally, longitudinal studies could better capture the long-term effects of physical activity on cardiovascular outcomes [65]. The potential of emerging technologies, such as wearable fitness trackers and virtual reality-based exercise programs, to enhance physical activity in older populations also warrants further investigation [66].

A key strength of our study is its focus on the unique context of Riyadh, Saudi Arabia, where environmental and cultural factors distinctly influence physical activity behaviors. For instance, the region’s high ambient temperatures limit opportunities for outdoor exercise, particularly during the extended summer months. Additionally, the built environment in Riyadh often lacks age-friendly infrastructure such as safe, accessible parks and pedestrian-friendly pathways, which further discourages regular physical activity among older adults. Cultural factors also play a significant role; for example, traditional norms may restrict women’s participation in public exercise, necessitating targeted interventions that consider privacy and gender-specific needs. Based on these findings, local policy recommendations should include the development of indoor recreational facilities, subsidized fitness programs for older adults, and urban planning initiatives that incorporate age-friendly designs. Tailored public health campaigns that address cultural sensitivities and promote active lifestyles among both men and women are also crucial.

Future research should build upon these results by incorporating longitudinal study designs to elucidate the temporal relationship between physical activity and cardiovascular outcomes. Moreover, a more comprehensive evaluation of nutritional habits—such as protein and micronutrient intake—should be integrated, given their impact on muscle mass and overall health. Objective measures of body composition, such as bioimpedance analysis, could provide a more accurate assessment of obesity and sarcopenia than BMI alone. Furthermore, studies exploring the use of wearable technology and virtual exercise interventions may offer innovative strategies to enhance physical activity levels among older adults in Saudi Arabia, particularly in a context where environmental and cultural barriers are prominent.

### Limitations

This study has several limitations that should be acknowledged. First, the cross-sectional design precludes establishing causality between physical inactivity and cardiovascular health outcomes. Longitudinal studies are needed to confirm the directionality of these associations. Second, the study relied on self-reported physical activity data, which may be subject to recall bias and social desirability bias. Although the Physical Activity Scale for the Elderly (PASE) is a validated tool, inaccuracies in reporting could influence the findings.

Additionally, the study was conducted at a single tertiary hospital in the Riyadh region, which may limit the generalizability of the results to other populations, particularly those in rural areas or other cultural contexts. The sample size, although sufficient for statistical analysis, may not capture the full diversity of older adults, including those with severe physical or cognitive impairments who were excluded from participation.

Lastly, while the study accounted for key cardiovascular risk factors such as hypertension, diabetes, and obesity, other potential confounders, such as dietary patterns, medication use, and genetic predisposition, were not examined. Future studies should incorporate these variables to provide a more comprehensive understanding of the relationship between physical inactivity and cardiovascular health.

## 5. Conclusions

This study highlights a significant association between physical inactivity and adverse cardiovascular health outcomes among older adults in Riyadh, Saudi Arabia. Participants with lower physical activity levels exhibited higher rates of hypertension, diabetes, obesity, and hyperlipidemia, reinforcing existing evidence on the role of physical inactivity in cardiovascular risk. A dose–response relationship was observed, with greater frequency, duration, and intensity of physical activity correlating with improved cardiovascular profiles. The study also identifies regional factors that may influence physical activity patterns, including high ambient temperatures, limited age-friendly infrastructure, and cultural attitudes that may restrict engagement in structured exercise, particularly among women. These findings underscore the importance of addressing environmental and social barriers to physical activity in this population.

While this study provides valuable insights, its cross-sectional design prevents establishing causality. Future research should focus on longitudinal studies to better understand the temporal relationships between physical activity and cardiovascular outcomes. Additionally, incorporating nutritional assessments, bioimpedance analysis, and wearable technology could enhance the accuracy of physical activity and health measurements in aging populations. In conclusion, these findings contribute to the growing body of evidence linking physical inactivity to cardiovascular health in older adults, particularly within the unique context of Riyadh. Continued research and tailored interventions are necessary to further explore and address the complex determinants of physical activity in this region.

## Figures and Tables

**Table 1:** Demographic characteristics of participants.

Characteristic	Categories	*n* (%)
Age (years)	60–64	64 (38.1%)
	65–69	55 (32.5%)
	70–74	31 (18.4%)
	≥75	18 (11.0%)
Gender	Male	91 (54.2%)
	Female	77 (45.8%)
Education	No formal	46 (27.3%)
	High school	71 (42.5%)
	College	51 (30.2%)
Marital Status	Married	119 (71.1%)
	Widowed	49 (28.9%)
Employment Status	Retired	105 (62.5%)
	Employed	26 (15.3%)
	Unemployed	37 (22.2%)
Smoking Status	Current	43 (25.8%)
	Former	58 (34.7%)
	Never	67 (39.5%)
Household Income	<10,000 SAR	79 (47.3%)
	10,000–20,000 SAR	59 (35.2%)
	>20,000 SAR	29 (17.5%)

**Table 2:** Physical activity levels based on PASE score.

Physical Activity Level	*n* (%)	Mean PASE Score ± SD	Walking Duration (min/day) ± SD	Vigorous Exercise (%)
Low	87 (51.8%)	42.7 ± 12.3	15.2 ± 7.1	12.3
Moderate	49 (29.2%)	85.4 ± 10.6	32.8 ± 10.4	34.6
High	32 (19.0%)	120.3 ± 15.1	52.1 ± 12.7	58.7

**Table 3:** Cardiovascular risk factors by physical activity level.

Risk Factor	Low Activity (%)	Moderate Activity (%)	High Activity (%)	*p*-Value
Hypertension	78.2	63.1	41.8	<0.001
Diabetes	64.4	49.8	28.7	<0.001
Obesity (BMI ≥ 30)	51.3	39.6	22.3	<0.001
Hyperlipidemia	69.0	55.3	35.4	<0.001
History of Stroke	12.6	8.4	4.3	<0.05
Coronary Artery Disease	28.7	18.5	9.8	<0.001

**Table 4:** Mean cardiovascular health indicators.

Indicator	Low Activity	Moderate Activity	High Activity	*p*-Value
Systolic BP (mmHg)	148.7 ± 9.4	132.6 ± 8.5	125.4 ± 7.2	<0.001
Diastolic BP (mmHg)	92.3 ± 7.1	85.4 ± 6.2	80.1 ± 5.6	<0.001
Total Cholesterol (mg/dL)	236.5 ± 18.3	202.4 ± 15.8	182.7 ± 12.9	<0.001
HDL Cholesterol (mg/dL)	38.2 ± 5.1	46.7 ± 6.4	52.3 ± 7.2	<0.001
LDL Cholesterol (mg/dL)	157.3 ± 14.8	128.9 ± 12.7	112.4 ± 10.3	<0.001
Blood Glucose (mg/dL)	161.7 ± 21.4	140.2 ± 18.5	121.5 ± 15.3	<0.001

**Table 5:** Predictors of cardiovascular disease (multivariate logistic regression).

Variable	Adjusted OR (95% CI)	*p*-Value
Physical Inactivity	1.98 (1.53–2.56)	<0.001
Hypertension	1.73 (1.34–2.23)	<0.001
Diabetes	1.52 (1.21–1.91)	<0.001
Obesity	1.41 (1.12–1.79)	<0.001
Smoking	1.29 (0.98–1.68)	0.045
Model Effect Size	R^2^ = 0.45	

## Data Availability

Data are available within the manuscript.
